# Abstractions for DNA circuit design

**DOI:** 10.1098/rsif.2011.0343

**Published:** 2011-07-20

**Authors:** Matthew R. Lakin, Simon Youssef, Luca Cardelli, Andrew Phillips

**Affiliations:** 1 Microsoft Research, 7 JJ Thomson Avenue, Cambridge CB3 0FB, UK; 2 Department für Physik, Lehrstuhl Rädler, Ludwig-Maximilians-Universität, Geschwister-Scholl-Platz 1, 80539 München, Germany

**Keywords:** DNA strand displacement, abstraction, modularity, formal methods, DNA oscillator

## Abstract

DNA strand displacement techniques have been used to implement a broad range of information processing devices, from logic gates, to chemical reaction networks, to architectures for universal computation. Strand displacement techniques enable computational devices to be implemented in DNA without the need for additional components, allowing computation to be programmed solely in terms of nucleotide sequences. A major challenge in the design of strand displacement devices has been to enable rapid analysis of high-level designs while also supporting detailed simulations that include known forms of interference. Another challenge has been to design devices capable of sustaining precise reaction kinetics over long periods, without relying on complex experimental equipment to continually replenish depleted species over time. In this paper, we present a programming language for designing DNA strand displacement devices, which supports progressively increasing levels of molecular detail. The language allows device designs to be programmed using a common syntax and then analysed at varying levels of detail, with or without interference, without needing to modify the program. This allows a trade-off to be made between the level of molecular detail and the computational cost of analysis. We use the language to design a buffered architecture for DNA devices, capable of maintaining precise reaction kinetics for a potentially unbounded period. We test the effectiveness of buffered gates to support long-running computation by designing a DNA strand displacement system capable of sustained oscillations.

## 1. Introduction

Biomolecular computers have great potential for use in intelligent nanomedicine. They allow computation to be performed at the molecular scale, while also interfacing directly with the molecular components of living systems. Nucleic acids are particularly suited for implementing biomolecular computers. They form stable structures that can be inserted into cells, and interactions between species can be precisely controlled *in vitro* by modifying their nucleotide sequences. The feasibility of using nucleic acids to solve computational problems was demonstrated by Adleman [1], who used DNA to solve an instance of the directed Hamiltonian path problem. Recent work has also highlighted novel therapeutic applications for nucleic acid computers, such as selectively triggering cell death in cancer cells [2].

As the cost of DNA synthesis continues to decrease [3], significantly more complex DNA computing devices are being constructed [4,5]. As a result, such devices are also becoming increasingly difficult to design by hand, to the point where design automation tools will soon be indispensable. Such tools should allow for modular designs that encapsulate particular motifs, which can be parametrized and easily replicated [6,7]. They should also allow some of the underlying complexity of the molecular interactions to be abstracted away when focusing on high-level design questions, since complex models are more difficult to work with and more computationally expensive to analyse. Once a high-level design has been completed, such tools should allow further complexity to be subsequently reintroduced, in order to obtain a more realistic model of the system's behaviour prior to its physical construction. In this paper, we present a programming language for designing DNA circuits, which meets these criteria.

Various approaches have been used to implement DNA circuits, some of which rely on ingredients such as restriction enzymes [8,9] or additional transcription machinery [10] to operate on the DNA strands. *DNA strand displacement* [11] is an alternative approach that relies solely on hybridization between complementary nucleotide sequences to perform computational steps. Strand displacement has been used with a wide variety of DNA structures, from simple linear complexes [12] and hairpins [13,14] to more sophisticated systems such as molecular walkers [15]. Strand displacement systems are driven by increases in entropy (from releasing strands) [12] and enthalpy (from forming additional base pairs), with irreversible reactions providing the thermodynamic bias towards producing output.

**Figure 1.**
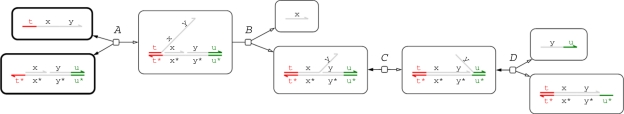
An example of toehold-mediated strand displacement. Edges with just a hollow arrowhead indicate irreversible reactions, whereas those which also have a solid arrowhead on the other end denote reversible reactions.

The reaction graph in figure 1 illustrates the strand displacement paradigm in action. The letters represent *domains*, which are finite, non-empty sequences of nucleotides. The domain x* represents the complementary domain, which will bind with x, constructed using Watson–Crick (C–G, T–A) complementarity. The grey domains are assumed to be sufficiently long that they bind irreversibly, while the coloured domains are assumed to be sufficiently short that they bind reversibly. We refer to these short domains as *toeholds*. We also assume that distinct letters represent distinct nucleotide sequences that do not interfere with each other.

Since toeholds bind reversibly, they are ideal for controlling interactions between species. Working from left to right in figure 1, in the first reaction (*A*), the toehold t in the single-stranded molecule binds reversibly to the exposed toehold t* in the double-stranded complex. This produces a double-stranded complex with an overhanging single strand. Since the x domain in the overhanging strand matches the x domain of an already bound strand, the junction performs a random walk along the x domain, called a *branch migration*. Eventually, the overhanging strand completely displaces the bound strand (*B*). Since x is not a toehold, we assume that the newly bound strand will not spontaneously unbind, which effectively renders this step irreversible. Following this, there is a reversible branch migration involving the y domain (*C*). Once the branch migration reaches the far right, the bound strand is only attached by the short toehold domain u and can therefore unbind (*D*). This basic computational mechanism allows us to construct computational devices, which translate input signals into output signals. Since the inputs and outputs are both just single strands of DNA, these devices can be combined to produce cascades that implement more complicated functionality [12].

In this paper, we expand on previous work [16] and present a language for designing modular DNA circuits using strand displacement. We provide a number of different abstractions for the language, which range from the simplified to the highly detailed. Moving between these different abstractions allows the user to model their system at a number of different levels of detail, which correspond to different levels of complexity. The various semantic abstractions that we describe encode different qualitative and quantitative assumptions about the behaviour of the system under experimental conditions. As an example, we use the language to design a novel architecture of *buffered gates* and compare simplified and detailed models of the system. Buffered implementations of reaction gates support long-running, potentially unbounded computations at fixed rates, which allows for robust encodings of chemical kinetics into DNA. As such, they offer an alternative to using a complex laboratory equipment such as continuous-flow reactors to deliver additional reactants [17]. We use these gates to design a three-phase oscillator in DNA, which displays sustained oscillations with precise kinetics.

Our implementation of the DNA Strand Displacement language (DSD) is available as a webserver at http://lepton.research.microsoft.com/webdna. The software allows a description of a DNA strand displacement system to be compiled into a reaction network for subsequent analysis and simulation. The user first programs a collection of DNA species, and the DSD compiler then automatically generates the reaction network corresponding to the different ways in which these species can interact with each other over time, including new species that can be produced as a result of the interactions. For example, for the simple system in figure 1, the user first programs the two DNA species outlined in bold, and the compiler then generates the complete reaction network derived from these species. The generated reaction network can then be simulated either stochastically or deterministically within the DSD tool itself, or exported in SBML format for simulation using a third-party tool such as COPASI [18]. The DSD tool was used to design and analyse the buffered gates and oscillator systems presented in this paper.^1^

## 2. Language definition

We define the set of possible configurations of DNA species by means of a formal *syntax*, and the set of possible interactions between species by means of a formal *semantics*. The definitions are given in the style of process calculi such as the pi-calculus [19,20], and are used both for implementing the DSD language and for reasoning about its properties.

### 2.1. Syntax

Species in the DSD language can be single-stranded molecules or double-stranded complexes. A single strand has an *orientation* from its 5′ end to its 3′ end, indicated by an arrow at the 3′ end. A double strand is formed of two single strands with opposing orientations and complementary nucleotide sequences, assuming Watson–Crick complementarity (C–G, T–A). For consistency, one of the strands is drawn on top and is oriented to the right, while its complementary strand is drawn underneath and is oriented to the left.

The syntax of the DSD language is defined in terms of *domains* M and *domain concatenations* S, L, R. A domain M represents a nucleotide sequence with explicit information about its orientation. For example, the domain 5′-CACACA-3′ denotes the sequence CACACA oriented to the right. This can also be written as 3′-ACACAC-5′, which denotes the same sequence oriented to the left. A domain can be a *long domain* N or a *short domain* N^. Short domains are assumed to be between 4 and 10 nucleotides in length and are also known as *toeholds*, while long domains are assumed to be at least 20 nucleotides in length. The intention is that toeholds are sufficiently short to bind reversibly whereas long domains are sufficiently long to bind irreversibly. We abbreviate a toehold N^ to N and use a different colour for each distinct toehold. In general, we assume that distinct domains are mapped to distinct, non-interfering nucleotide sequences using established coding techniques [12,21]. Thus, domains allow us to abstract away from the underlying nucleotide sequences that occur in a physical implementation. A domain concatenation S consists of finitely many domains with the same orientation, where domain concatenations L and R can potentially be empty, written (_). The *complement* M* of a domain M is the domain that hybridizes with M. This is computed by reversing the orientation of the domain and taking the Watson–Crick complement of each individual nucleotide in the domain. For example, the complement of 5′-CACACA-3′ is 3′-GTGTGT-5′, which can also be written as 5′-TGTGTG-3′. Similarly, the complement S* of a domain concatenation S is computed by reversing then taking the complement of each domain in S.

The syntax of the DSD language is defined in table 1 in terms of *systems* D, which consist of species in solution. A single species can be a *strand* A or a *gate* G. A strand can be written either as an *upper* strand <S>, which denotes a concatenation of domains S oriented to the right, or as a *lower* strand {S}, which denotes a concatenation of domains S oriented to the left. Since nucleic acids may take different physical orientations in three-dimensional space, the distinction between upper and lower strands is purely syntactic, serving only as a convenient way of presenting strands on the page. We, therefore, assume that species are equal up to rotation symmetry, where *rotate*(*I*) denotes the result of rotating the strand or gate *I* through 180°. Formally, we identify individual species *I* up to a *structural equivalence* relation (≡) which is such that *I* ≡ *rotate*(*I*) (see electronic supplementary material for definitions). For example, the textual and graphical representations of two strands that are equivalent up to rotation symmetry are shown below, along with a possible assignment of nucleotide sequences to their domains.


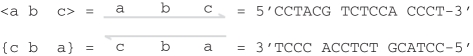


**Table 1.** Syntax of the DSD language, in terms of strands A, gates G and systems D. Where present, the graphical representation below is equivalent to the program code above.

syntax		description
A	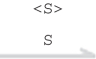	upper strand with domain concatenation S
	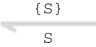	lower strand with domain concatenation S
G	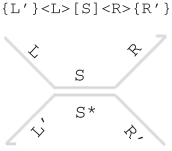	double-stranded complex [S] with overhanging single strands <L>, <R> and {L'}, {R'}
	G1:G2	gates joined along a lower strand
	G1::G2	gates joined along an upper strand
D	A	strand A
	G	gate G
	D1 | D2	parallel systems D1, D2
	new N D	system D with private domain N
	X(ñ)	module X with parameters ñ

A *double strand* [S] denotes an upper strand <S> bound to its complementary lower strand {S*}. Only the domain concatenation S of the upper strand is written explicitly, enabling a more compact notation. A gate G can be a double-stranded complex {L'}<L>[S]<R>{R'}, which consists of a double stranded region [S] with overhanging strands <L>,<R> and {L'},{R'}. This represents an upper strand <L S R> bound to a lower strand {L' S* R'} along the double-stranded region [S]. We omit empty overhanging strands <_> and {_} for convenience. A gate can also be a concatenation G1:G2 of two gates that share a common lower strand, or a concatenation G1::G2 of two gates that share a common upper strand. We represent the joining of two gates explicitly in the graphical syntax. For example, the gate {a}<b>[c]<d>{e}:{v}<w>[x]<y>{z} is represented as follows, by joining the left and right gates along their lower strands between the e and v domains.


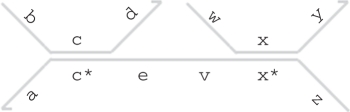


In general, there may be several equivalent ways to represent the joining of two gates in the DSD syntax. This arises from the fact that an overhanging strand joining two gates can belong either to the left or the right gate. For example, [a]{b}:[c] and [a]:{b}[c] represent the same gate, as do [a]<b>::[c] and [a]::<b>[c]. We formalize this by including rules for gates with shared overhangs in the structural equivalence relation mentioned above (see electronic supplementary material).

Multiple systems D1,D2 can be present in parallel, written D1|D2. We abbreviate K parallel copies D|..|D of a system D to K*D. A domain N can also be *restricted* to the system D, written new N D. This represents the assumption that the domains N and N* do not appear outside of D, which is a useful programming abstraction. We also allow module definitions of the form X(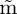)=D, where 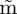 is a list of module parameters and X(ñ) is an instance of the system D with the parameters 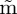 replaced by values ñ. We assume a fixed environment *E* of module definitions, which are declared at the start of the program. The definitions are assumed to be *non-recursive*, meaning that a module cannot invoke itself, either directly or indirectly via another module. We also assume that systems D are well-mixed, and formalize this by extending the structural equivalence relation (see electronic supplementary material).

Finally, we define a notion of *well-formed* systems, where a system is well-formed if no long domain and its complement are exposed simultaneously. This ensures that two species can only interact via complementary toeholds, as discussed in Zhang *et al.* [12]. In the remainder of this paper, we assume that all systems are well-formed.

### 2.2. Semantics

**Figure 2.**
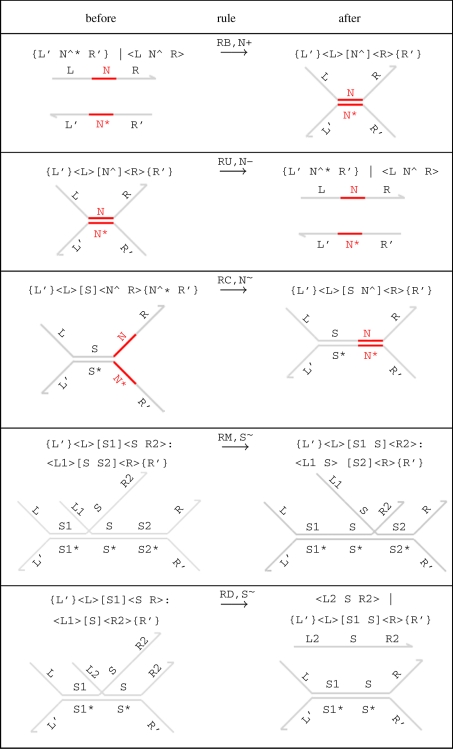
Elementary reduction rules of the DSD language. For each rule, the graphical representation below is equivalent to the program code above. We let S^∼^ denote the migration rate of a domain concatenation S, and we let N^+^ and N^−^ denote the binding and unbinding rates, respectively, of a toehold N^. We let fst(S) and lst(S) denote the first and last domains in a concatenation S, respectively, and we assume that fst(R2) ≠ fst(S2) for rule (RM). This ensures that branch migration is maximal along a given domain concatenation and that rules (RM) and (RD) are mutually exclusive.

We formalize the different ways in which species can interact with each other by defining a set of elementary *reduction rules* (figure 2). The rules are of the form 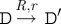, which states that D can *reduce* to D' by performing an interaction with finite rate *r* according to rule *R*. Rules (RB) and (RU) define strand binding and unbinding on a toehold, where each toehold N^ is associated with corresponding binding and unbinding rates given by N^+^ and N^−^, respectively. This relies on our assumption that toeholds are sufficiently short that they hybridize reversibly. There is no rule for binding on a long domain, since the well-formedness constraints prevent a long domain and its complement from being exposed simultaneously. There is also no rule for unbinding on a long domain, since the hybridization of two long domains is assumed to be sufficiently strong that it is essentially irreversible. Rule (RC) defines a toehold covering reaction, where an exposed toehold in a lower strand is covered by a complementary exposed toehold in an upper strand. We assume that covering reactions are also irreversible, since they result in the formation of a long double-stranded complex, which is thermodynamically stable. Rule (RM) defines a branch migration reaction, where one strand partially replaces another on a gate. Each domain concatenation S is associated with a corresponding branch migration rate S^∼^, which depends on the number of domains. The branch migration rate for a domain of length *L* is given by *r*/*L*^2^, where *r* is the single nucleotide migration rate, taken to be 8000 s^−1^ [22]. The rule models a maximal sequence of elementary branch migration steps as a single reduction. Rule (RD) defines a strand displacement reaction that results from a branch migration, where one strand completely displaces another from a gate.

The elementary reduction rules of figure 2 can also occur inside a range of contexts. We formalize the set of possible contexts by defining additional contextual reduction rules (see electronic supplementary material). For example, if we join additional double-stranded gates to either side of a reacting complex, we still obtain a valid reaction. This is illustrated by the following reaction, which is derived from the elementary reduction rule (RD) using the contextual rules.


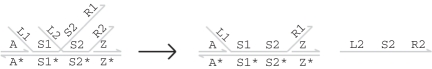


We also use contextual rules to express that reductions can take place on either the top or bottom strand, or towards the left or right side of the gate. For example, rule (RD) can also be used to displace a strand by migrating towards the left-hand side of a gate, by application of a contextual symmetry rule. The combination of elementary and contextual reduction rules allows interactions involving at most two individuals. An important point to note is that the set of reduction rules can be readily extended as needed, to incorporate additional assumptions about the nature of interactions between species.

As described previously, a key assumption of the language is that two species can only interact with each other via complementary toeholds. This is enforced syntactically by our notion of well-formed systems. In order to ensure that species can only ever interact on toeholds, it is sufficient to show that this well-formedness property is preserved by reduction. The proof is by case analysis on the various reduction rules (see electronic supplementary material). The key fact is that none of the rules result in the exposure of a long domain, which was not previously exposed.

### 2.3. A hierarchy of semantics

The main contribution of this paper is to equip the DSD language with multiple semantic interpretations that abstract away some of the complexity of the DNA interactions. We achieve this by parametrizing the semantics of the DSD language, so that model accuracy can be balanced with the computational cost of model analysis. The resulting modifications give rise to a hierarchy of semantics for the language. This allows a system to be formalized once and then analysed under many different behavioural assumptions. Below, we describe the specific behaviours we might like to abstract away or introduce, namely *unproductive*, *leak* and *fast* reactions.

#### 2.3.1. Unproductive reactions

Some of the reactions involving a given collection of species are *unproductive* in the sense that they do not contribute meaningfully to the progress of a simulation. An example of an unproductive reaction is the case where a strand binds to a gate along a short domain, but cannot initiate any subsequent migration or displacement reactions, as illustrated below.


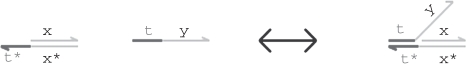


**Figure 3.**
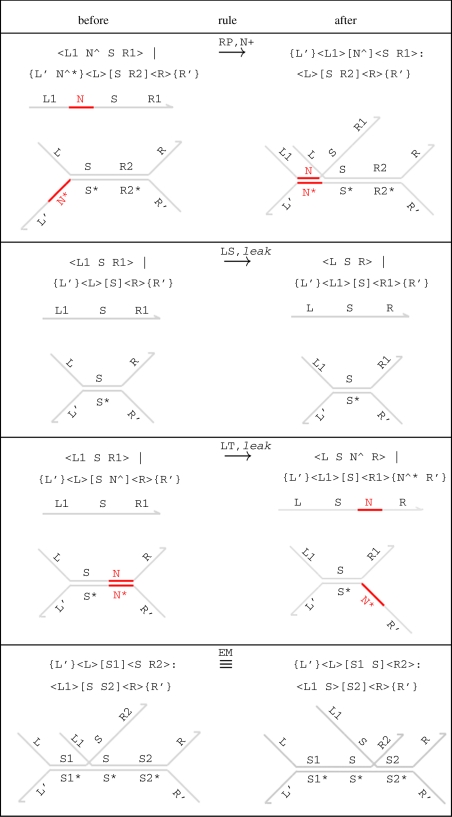
Additional reduction rules for the DSD language. Rule (RP) ensures that toehold binding can only take place if a subsequent migration reaction is possible, while rules (LS) and (LT) model interferences between species, assuming that S ≠ N^ in rule (LS) and R1 ≠ N^ R1' in rule (LT). Finally, rule (EM) ensures that gates are equivalent up to branch migration.

In our formalism, a binding reaction is considered to be unproductive, if none of the domains immediately adjacent to the binding toehold on the stand is complementary to those on the gate. *Productive* reactions are defined by rule (RP) in figure 3.

#### 2.3.2. Leak reactions

Our key assumption is that short domains are short enough to bind reversibly, while long domains are long enough to bind irreversibly. However, in practice, a long domain does not need to unbind completely in order to initiate a reaction: a double-stranded region of DNA may start to fray slightly at one end, creating a short exposed region of single-stranded DNA, which can then function as a temporary toehold. A free strand with the same domains as one of the bound strands can then bind to this exposed toehold and displace the bound strand. Such reactions are referred to as *leaks*. Leak reactions in DSD are defined by rules (LS) and (LT) in figure 3. Since leaks occur with very low probability, we assume that the leak rate *l* is much smaller than the rates of other types of reactions [23]. If there is a mismatched toehold at one end of the double-stranded segment, then the other end must unbind in order to initiate the leak reaction, as in rule (LT). We do not include a rule for leak reactions with mismatched toeholds at both ends, since we assume that the rate of such a reaction is negligible.

Leak reactions represent a form of unwanted interference between species, which can sometimes have a significant impact on the behaviour of the system. However, allowing leaks can drastically increase the number of possible reactions in the system, leading to a sizeable increase in the computational cost of analysis. Nevertheless, it is important that tools for designing DNA circuits allow unwanted interference to be modelled and studied formally, so that its effects on the overall behaviour of the system can be quantified.

#### 2.3.3. Fast reactions

Some of the reactions in our models occur on a much faster timescale than others. In order to simplify the model and improve the efficiency of simulation, it is often useful to abstract away from these *fast reactions*. In some cases, we may decide to treat fast reactions as if they happen instantaneously; in others, we may merge them into a single step with a fixed rate. In what follows we distinguish between fast reactions and other reactions, which we refer to as *slow reactions*. The exact definitions of which reactions are fast and which are slow, and the value of the fast reaction rate, will depend on our chosen semantic abstraction.

Note that in the general case, a given system D can potentially reduce to multiple possible systems D' through mutually exclusive, competing fast reactions. However, if we ensure that the initial system does not have any competing fast reactions, then we can show that no subsequent species produced by the system will have competing fast reactions (see electronic supplementary material).

### 2.4. Semantic abstractions

We now present a formal definition of the hierarchy of semantic abstractions available in the DSD language.

**Definition 2.1.** A semantic abstraction *σ* is a triple (*s*,*l*,*u*) where *s* *ε* {*Infinite, Default, Finite, Detailed*} denotes the *level* of abstraction, *l* *ε* {true,false} denotes whether or not to include leaks, and *u* *ε* {true,false} denotes whether or not to include unproductive reactions. If *σ* = (*s,l,u*), we say that *level*(*σ*) = *s*, that *leaks*(*σ*) = *l* and that *unproductive*(*σ*) = *u*.

Each of the four levels of abstraction is defined in terms of a set of slow reduction rules *slowrules*(*σ*) and a disjoint set of fast reduction rules *fastrules*(*σ*) (table 2), chosen from the set of primitive reduction rules of figures 2 and 3. These sets also depend on whether the semantic abstraction *σ* includes leaks or unproductive reactions. If *leaks*(*σ*)=true, then we add the leak rules (LS) and (LT) to the set of slow reduction rules. In the *Detailed*, *Finite* and *Default* semantics, we use the value of *unproductive*(*σ*) to decide whether to include unproductive reactions: if *unproductive*(*σ*)=false, then we add the productive binding rule (RP) to the set of slow reductions; otherwise, we use the more general (RB) rule which may include unproductive binding reactions. The exception to this is the *Infinite* level of abstraction, where we use (RP) irrespective of the value of 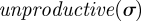. The levels of abstraction also vary in the way in which fast reduction rules are merged together, and the way in which branch migration is handled (table 3).

**Table 2.** Semantic abstractions for the DSD language. For a given semantic abstraction *σ*, the table presents the primitive reduction rules from figures 2 and 3, which correspond to slow reductions *slowrules*(*σ*), and fast reductions *fastrules*(*σ*). These vary depending on the level of abstraction *level*(*σ*). The values of *unproductive*(*σ*) and *leaks*(*σ*) influence which rules appear in *slowrules*(*σ*). We define *leakrules*(*σ*)=(**if** *leaks*(*σ*) **then** {LS, LT} **else** {}) and *bindrules*(*σ*)=(**if** *unproductive*(*σ*) **then** {RB} **else** {RP}). Glossary of rules: (RM) branch migration, (RD) strand displacement, (RU) toehold unbinding, (RC) toehold covering, (RB) toehold binding, (RP) productive toehold binding, (LS) and (LT) leaks.

*level* (*σ*)	*slowrules* (*σ*)	*fastrules* (*σ*)
*Detailed*	*bindrules*(*σ*) ∪ *leakrules*(*σ*) ∪ {RM, RD, RU, RC, RF}	{}
*Finite*	*bindrules*(*σ*) ∪ *leakrules*(*σ*)	{RD, RC, RU}
*Default*	*bindrules*(*σ*) ∪ *leakrules*(*σ*) ∪ {RU}	{RD, RC}
*Infinite*	{RP} ∪ *leakrules*(*σ*)	{RD, RC, RU}

**Table 3.** Reaction merging rules for the DSD language. Depending on the semantic abstraction *σ*, fast reactions may be merged with slow reactions or treated as separate reactions in their own right. The top two rules define fast and slow reductions for a given semantic abstraction *σ* according to table 2. The next rule formalizes the inclusion of branch migration in the structural equivalence relation ≡_*σ*_ when *level*(*σ*) ≠ *Detailed*. The remaining rules define the merged reduction relation (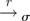) in terms of the fast and slow reduction relations.

conditions	before	rule	after
*Rεslowrules*(*σ*) **and** 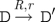	D	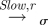	D′
*Rεfastrules*(*σ*) **and** 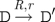	D	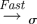	D′
*level*(*σ*) ≠ *Detailed***and** 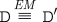	D	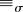	D′
*level*(*σ*) = *Detailed***and** 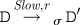	D	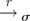	D′
*level*(*σ*) = *Finite***and** 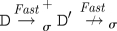	D	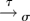	D′
*level*(*σ*) = *Finite***and** 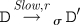	D	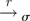	D′
*level*(*σ*) = *Default***and** 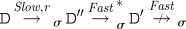	D	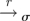	D′
*level*(*σ*) = *Infinite***and** 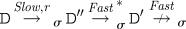	D	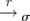	D′

We formalize the notions of fast and slow reductions as follows. We write 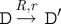 to mean that the system D can be reduced to D′ in a single step at rate *r*, using one of the elementary reduction rules *R* from figures 2 and 3, and the additional contextual rules from the electronic supplementary material. We then derive a fast reduction relation 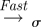, parametrized by the semantic abstraction *σ* (table 3). This rule picks out any reduction that uses an elementary rule *R* from the set *fastrules*(*σ*) and casts it into the fast reduction relation. We write 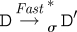 if D reduces to D' by a sequence of zero or more fast reductions, 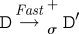 if that sequence is non-empty, and 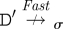 if D' cannot perform any fast reductions at all. Similarly, we derive a parametrized slow reduction relation 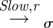 corresponding to a reduction using an elementary slow reduction rule from *slowrules*(*σ*) (table 3). The reduction is labelled with the corresponding reaction rate *r*.

In order to abstract away from fast reactions, we need some way of *merging* fast reactions with each other and with the slow reactions. Table 3 presents inference rules for deriving a merged reduction relation (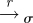) from the fast (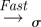) and slow (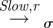) reduction relations. The details depend on the level of abstraction chosen (*level*(*σ*)), as summarized below.

#### 2.4.1. Detailed abstraction

There are no fast reductions, and every slow reduction 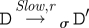 corresponds to a single reduction 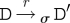. As a result, toehold binding, unbinding, strand displacement and branch migration all appear as distinct reactions. This is the most detailed of the four levels of abstraction and is valid in the limit of high concentration, when toehold binding rates are sufficiently high that the duration of intermediate steps (such as branch migration) can no longer be discounted. For example, in the case of toehold exchange reactions [22], our *Detailed* abstraction corresponds to the ‘three-step model’ from that paper.

#### 2.4.2. Finite abstraction

We assume that strand displacement (RD), toehold covering (RC) and toehold unbinding (RU) are all fast reductions. As in the *Detailed* case, we assume that every slow reduction 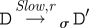 corresponds to a single reduction 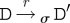. In addition, a maximal sequence of one or more fast reductions 
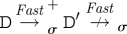
 is combined into a single merged reduction with finite rate *τ*. This models fast reductions as taking a finite amount of time, while abstracting away from individual fast reductions.

#### 2.4.3. Default abstraction

We assume that strand displacement (RD) and toehold covering (RC) are fast reductions that are effectively instantaneous. As a result, if a slow reduction 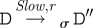 is followed by a maximal sequence of zero or more fast reductions 
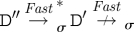
 then these are merged into a single reduction 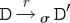. Note that the sequence of fast reductions may be empty, resulting in the reduction 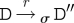. This corresponds to the semantics originally presented in Phillips & Cardelli [16].

#### 2.4.4. Infinite abstraction

We assume the same set of fast reductions as in the *Finite* case and the same rule for merging fast reductions, but fast reductions are instantaneous in the *Infinite* abstraction. Since unbinding reductions are instantaneous, any strand that binds but cannot initiate a cover or displacement reaction will immediately disassociate—hence we ignore unproductive binding reactions. This model is valid in the limit of low concentration, when the rates for toehold binding reactions are sufficiently low that unary reactions are instantaneous in comparison. In the case of toehold exchange reactions, our *Infinite* abstraction corresponds to the ‘bimolecular reaction model’ from Zhang & Winfree [22].

For efficiency reasons, we also discard any merged reactions of the form 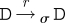, which we call *circular*. This is reasonable because we assume that all reaction rates are exponentially distributed, so ignoring circular reactions has no effect on the dynamics of the system.

For each of the levels of abstraction involving merged fast reactions (*Finite*, *Default* and *Infinite*), we treat gates that differ only by branch migrations as being equal. Since branch migration steps are reversible, treating species as equal up to branch migration greatly simplifies the task of merging fast reactions by removing the possibility of loops in fast reaction sequences. We achieve this by building branch migration into the definition of structural equivalence for the *Finite*, *Default* and *Infinite* semantic abstractions. Thus, the structural equivalence relation 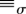 is parametrized by the semantic abstraction *σ*. Formally, if *level*(*σ*) ≠ *Detailed*, then the branch migration equivalence rule (em≡) from figure 3 is included in the definition of the structural equivalence relation 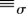.

**Figure 4.**
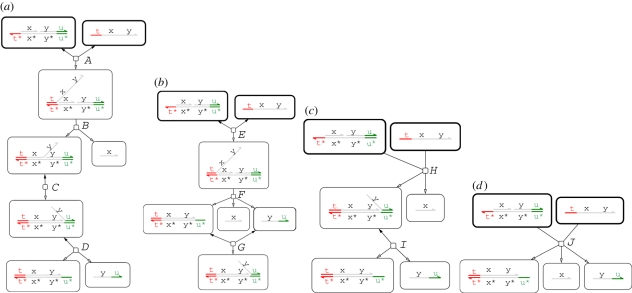
Example of the language hierarchy. The four images show the reaction graphs produced for the program (<tû x y> | {tû *}[x]:[y uû]) for the four possible values of *level*(*σ*), where *unproductive*(*σ*)=*leaks*(*σ*)=false. Edges with just a hollow arrowhead indicate irreversible reactions, whereas those which also have a solid arrowhead on the other end denote reversible reactions. The complexity of the reaction graphs decreases from left to right as we move from more detailed to more abstract semantic models of the DNA interactions.

Figure 4 illustrates our approach with a concrete example that shows how different reaction graphs can be produced from the same program by using different levels of abstraction. In the *Detailed* case, the initial strand and gate can bind reversibly (*A*) and the <x> strand can then be displaced (*B*). The <y u^> strand can then migrate backwards and forwards along the y domain (*C*), and subsequently unbind and bind reversibly on the u^ toehold (*D*). In the *Finite* case, following the binding of the initial strand and gate (*E*), the displacement of the <x> strand and the migration and subsequent unbinding of the <y u^> strand all take place in a single step (*F*). The <y u^> strand can subsequently bind and unbind reversibly on the u^ toehold (*G*). For *Default*, since strand displacement is instantaneous, the initial binding and displacement reactions take place in a single step (*H*). Furthermore, species are considered equal up to branch migration under this abstraction, so the branch on the top strand is migrated far as possible to the right by default. This strand can subsequently unbind and bind reversibly on the u^ toehold (*I*). At the *Infinite* level of abstraction, the binding, displacement, migration and unbinding reactions are all merged into a single reaction (*J*), where the binding reaction has a finite rate and the other reactions are instantaneous. Since both branch migration and unbinding reactions are instantaneous, the re-binding reaction of the <y u^> strand is not included, as this is a circular reaction.

### 2.5. Language compilation

In order to simulate a DSD program, we first compile the program to a set of chemical reactions, and then apply either a deterministic or stochastic simulation method (see electronic supplementary material). There are two modes of compilation, a *saturating* (SAT) mode and a *just-in-time* (JIT) mode.

In SAT mode, the compiler starts with an initial set of species and computes the set of possible reactions between those species, using the merged reduction relation 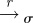, which depends on the choice of semantic abstraction *σ*. We use the 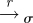 reduction relation to determine the reactions, since this allows us to present generic definitions of compilation and simulation for an arbitrary semantic abstraction *σ*. These reactions can potentially generate new species, which in turn can potentially generate new reactions. The SAT compiler continues generating reactions and species in this way until no new species can be generated. The result is the set of all possible reactions that can potentially take place given the initial set of species.

In contrast, the JIT compiler only computes the set of reactions that directly involve the initial species, and then uses these reactions together with the initial species populations to make a choice as to which reaction to select, according to standard stochastic simulation methods (see electronic supplementary material). The chosen reaction is then applied to the current set of species, resulting in some species being consumed and some new species being produced. The set of reactions involving the updated set of species is computed, and the next reaction is chosen as above. Thus, in JIT mode, the compilation of reactions is interleaved with the simulation of the system. This allows systems with potentially unbounded numbers of species and reactions to be simulated exactly. The JIT compiler is an extension of the generic compilation algorithm presented in Pauleve *et al.* [24]. Our contribution is to instantiate this generic algorithm to the DSD language and to define a novel use of the JIT compiler to explore all possible trajectories of the system, in order to generate a continuous time Markov chain (CTMC) representation (see electronic supplementary material).

## 3. Buffered reaction gates

In this section, we demonstrate the practical use of the DSD language to design DNA computational elements. We show how different levels of detail can be achieved using different semantic abstractions, and how analysis of DSD programs can be used as a starting point for formal verification of DNA circuit designs.

**Figure 5.**
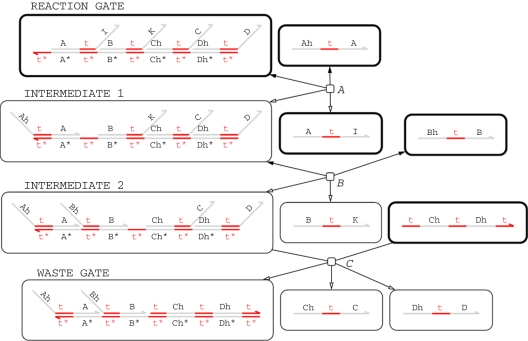
Chemical reaction network for two input, two output *unbuffered* join gate when *σ*=(*Infinite*,false,false). Edges with just a hollow arrowhead indicate irreversible reactions, whereas those that also have a solid arrowhead on the other end denote reversible reactions. Nodes with a thick black line denote species that are present initially.

Figure 5 presents the chemical reaction network (CRN) for a simple *unbuffered* implementation of a join gate, which accepts two input signals *A* and *B* and produces two output signals *C* and *D*. A signal *X* is represented as a three-domain strand of the form <hX t^ X>, where the choice of history domain hX is irrelevant. The initial reaction gate accepts the input strands <hA t^ A> (*A*) and <hB t^ B> (*B*). The ‘intermediate 2’ gate then accepts a <t^ Ch t^ Dh t^> fuel strand, which triggers the release of the output strands <Ch t^ C> and <Dh t^ D> (*C*). Note that this simple design does not include additional structures for garbage collection of waste species.

Unfortunately, using an unbuffered gate design such as this does not provide stable kinetics: gates are continuously being consumed during the operation of a DNA circuit, since the energy driving the circuit is partly provided by gates being turned into waste structures. Hence, the gate populations are not fixed, which means that the kinetics of the system changes over time. This can be particularly problematic for long-running computations. One solution is to use a very large and hence almost-constant population of gates with respect to the population of signals, but this means that the design of the system becomes linked to the length of time for which the system is intended to run. An alternative solution is to continually replenish the gates during the course of the computation in order to maintain the desired kinetics. However, unless the gates are replenished very frequently, for example, by means of a sophisticated experimental set-up, the kinetics will drift between replenishments, which may adversely affect the behaviour of the system. The optimal situation would, therefore, be to maintain a constant population of gates awaiting input.

An abstract buffering technique was proposed in Cardelli [25] to achieve constant concentrations of gates awaiting input. Here, we refine this technique and propose concrete structures that can be simulated using the DSD language. The idea is to maintain a quasi-constant but relatively low concentration of *initialized* gates by means of a higher concentration of *buffered* gates, which are initialized on demand. Only initialized gates implement the desired chemical reaction. Since changes in the population of buffered gates do not significantly affect the kinetics of the reactions, the buffered gates only need to be replenished periodically. Thus the effective rates of the reactions can be held constant for arbitrarily long periods of time, provided that the buffered gates are not completely exhausted. This approach is equivalent to using a continuous-flow reactor to provide auxiliary gates at low concentration, which was suggested in Soloveichik *et al.* [17] (see electronic supporting material) as a means of counteracting the effects of gate depletion and leaks. However, buffered gates achieve this effect based solely on the design of the nucleic acid sequences, without the need for additional laboratory equipment.

We will use a three-phase oscillator to illustrate the benefits of buffered reaction gates. Producing oscillatory behaviour in an experimental setting is non-trivial since it relies on precise kinetics being maintained over an extended period. This makes an oscillator system an ideal candidate for implementation using buffered gates. Figure 9 in §3.2 demonstrates the benefit of using buffered reaction gates to implement an oscillator. A direct comparison of the kinetics of individual buffered and unbuffered join gates is less illuminating, since the benefits of buffered gates are most apparent in systems with long-running computations (see electronic supplementary material).

More generally, the buffered gate scheme presented in this section can be used to construct a broad range of long-running dynamical systems in DNA. Each buffered gate BJ2x2(Buffer,Fuel,Init,A,B,C,D) effectively corresponds to a chemical reaction of the form 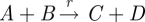, where the rate *r* is determined by setting the Init parameter. Thus, multiple instances of the gate can be used to construct an arbitrary chemical system consisting of reactions with two reactants and two products. Furthermore, simple modifications to the gate structure can be made to simulate reactions with different arities, using a similar approach to Soloveichik *et al.* [17].

### 3.1. Buffered gate implementation

**Figure 6.**
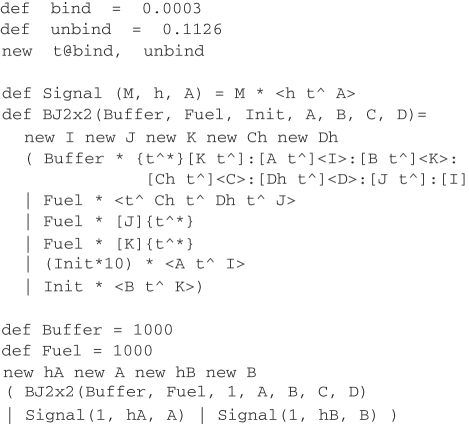
DSD code for buffered join gate.

We have designed a buffered join gate that accepts two inputs *A* and *B* and produces two outputs *C* and *D*, with an initial input that initializes a buffered gate, and an additional output that initializes another buffered gate to replace the gate that has been consumed. The DSD code for the buffered join gate, together with input strands representing *A* and *B*, is presented in figure 6. The Signal module represents a single-stranded signal and the BJ2x2 module represents the buffered join gate itself. The Buffer variable controls the initial population of buffered gates, Init controls the number of those gates that should be initialized at any one time and Fuel controls the number of fuels for displacing outputs and performing garbage collection, which should be present in excess. Note that by default, the DSD language assumes discrete populations of species and produces stochastic simulations. Thus, the units of the rate constants bind and unbind in figure 6 are s^−1^. See electronic supplementary material for details on converting discrete populations to continuous concentrations for deterministic simulations.

**Figure 7.**
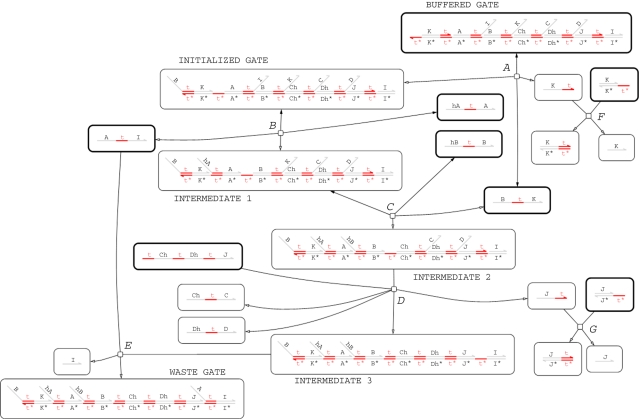
Chemical reaction network for two input, two output buffered join gate when *σ*=(*Infinite*,false,false). Edges with just a hollow arrowhead indicate irreversible reactions, whereas those that also have a solid arrowhead on the other end denote reversible reactions. Nodes with a thick black line denote species that are present initially.

The CRN for this program, generated using the *Infinite* semantic abstraction and the SAT compiler (ignoring leaks and unproductive reactions), is shown in figure 7. This design extends the simplified unbuffered gate design from figure 5 by adding additional reactions for buffering (*A*) and garbage-collection (*E*,*F*,*G*). Initially, the trigger strand <B t^ K> turns a buffered gate into an initialized gate (*A*), which can then accept the input strands <hA t^ A> (*B*) and <hB t^ B> (*C*). The ‘intermediate 2’ gate then accepts a <t^ Ch t^ Dh t^ J> fuel strand which triggers the release of the output strands <Ch t^ C> and <Dh t^ D> (*D*). The by-products of the reactions are garbage-collected, turning them into unreactive waste (*E*,*F*,*G*). The design of the gate ensures that the first input strand <hA t^ A> is not consumed irreversibly until there is also a corresponding <hB t^ B> input. This is vital for the correctness of the gate design because there might be another gate in the system which could make use of the <hA t^ A> strand. Crucially, consumption of the <hB t^ B> strand (*C*) causes the release of another trigger strand <B t^ K>, which initializes another buffered gate to replace the gate that was consumed. The long domains I, J, K, Ch and Dh are restricted to only appear in the scope of a particular instance of the module BJ2x2, which prevents unwanted crosstalk between gates. The intermediate product <A t^ I> is also included as an initial species, in order to prevent too many inputs binding to a particular reaction gate, which could disrupt the kinetics of the system. Empirical testing showed that using 10 copies of this strand for every initialized gate seems to produce reasonable results.

**Figure 8.**
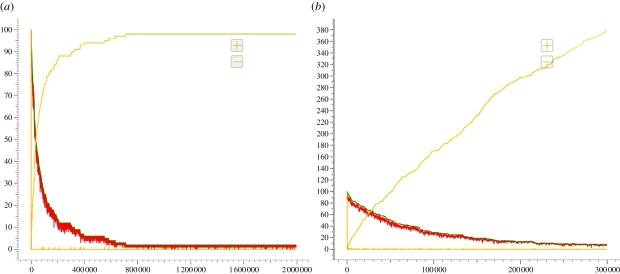
Stochastic simulation plots of 100 copies of the buffered join gate, both with (*b*) leak reactions and (*a*) without. The red and green lines are the populations of the <hA t^ A> and <hB t^ B> input strands, respectively. The blue and yellow lines are the population of the <Ch t^ C> and <Dh t^ D> output strands (the populations of these are always identical). The darker orange line is the population of the trigger strands <B t^ K>, which initialize buffered gates on demand. The plot with leaks enabled is qualitatively different from the plot without leaks, suggesting that the unwanted inference is adversely affecting the behaviour of the system (see §3.3 for further discussion on how leak reactions affect the buffered join gate).

Running a stochastic simulation for 100 copies of the join gate produces the plot shown in figure 8*a*. We see that, over time, the populations of the input strands tend to zero whereas the populations of the output strands tend to 100, as we would expect. The red line displays a jagged effect because <hA t^ A> strands which bind to the gates are often pushed back off by the <A t^ I> strands from the BJ2x2 module definition. As the buffered join gate is a relatively small system, it is feasible to construct the corresponding CTMC by computing all possible interleavings of the reactions from the CRN in figure 7, given a single copy of the input strands <hA t^ A> and <hB t^ B>. We find that the CTMC contains 30 states and 72 transitions. Furthermore, there is a single terminal state (that is, a state from which no further reactions are possible). This fact provides the first piece of evidence that our gate design functions as expected. See the electronic supplementary material for more information on the CTMC.

The CRN presented in figure 7 is a simplified approximation to the true behaviour of the system, because we used the *Infinite* semantics (and ignored unproductive reactions). In order to obtain a more realistic model of the system, we move to the other end of our spectrum of levels of abstraction, from *Infinite* to *Detailed*. This results in a larger CRN and a larger CTMC because the *Detailed* semantics includes separate reactions for branch migration, strand displacement and toehold unbinding, which were assumed to be instantaneous in the *Infinite* semantics. Table 4 shows how the sizes of the CRN and the CTMC increase as we move from the *Infinite* to the *Detailed* levels of abstraction (excluding leaks and unproductive reactions). Even in the *Detailed* semantics, there is still only one terminal state and it contains precisely the same species as the terminal state for the *Infinite* system. Thus, moving to the more fine-grained *Detailed* semantics has not affected the qualitative behaviour of the system (table 4).

**Table 4.** Metrics of system complexity for a single buffered join gate using different semantic abstractions. We fix *unproductive*(*σ*)=*leaks*(*σ*)=false and vary *level*(*σ*). We tabulate the numbers of species and reactions in the full CRN and the numbers of states and state transition arcs in the full CTMC of the system.

*level*(*σ*)	species	reactions	states	arcs
*Infinite*	22	10	30	72
*Default*	26	18	52	145
*Finite*	30	26	158	640
*Detailed*	35	35	231	954

### 3.2. A three-phase oscillator

We can compose three join gates into a circuit to obtain a three-phase oscillator. Given three signals *A*, *B* and *C*, the gates implement the reactions 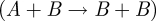, 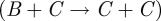 and 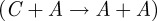 [26,27]. This is an intrinsically unstable oscillator but a very simple one. We simply reuse the previous definitions of signals (Signal) and the buffered join gate (BJ2x2) from figure 6, along with an initial population of signals to start the oscillation.


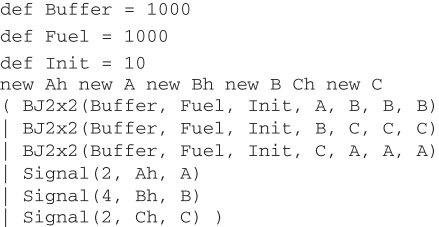


**Figure 9.**
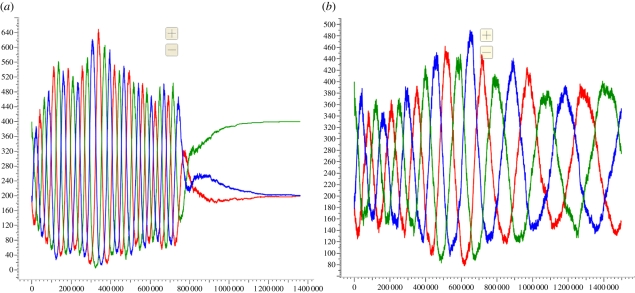
Stochastic simulation plots for the three-phase oscillator, comparing buffered and unbuffered implementations. (*a*) Buffered oscillator using the buffered join gate defined in figure 6. The initial populations of buffered gates, initialized gates and fuels were 10 000, 1000 and 100 000, respectively. We observe constant periods of oscillation right up until the buffered gates are completely depleted at around 700 000 time units. (*b*) Unbuffered oscillator using an unbuffered join gate without garbage collection, the CRN of which was presented in figure 5. The initial populations of gates and fuels were 10 000 and 100 000, respectively. The higher initial population of unbuffered gates compared with initialized gates in the buffered case is offset to an extent by the fact that more ‘backwards strands’ <A t^ I> are present in this case. We observe a gradual drift in the period of oscillation as the gate population is gradually depleted. (*a*,*b*) red line, sum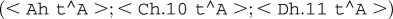; green line, sum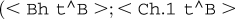; 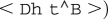; blue line, sum
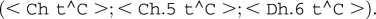

The CRN for the buffered oscillator program (using the *Infinite* semantics) contains 168 species and 264 reactions. The CTMC for this system is too large for analysis using currently available tools. However, we can run stochastic simulations to obtain plots of the three signals oscillating over time. Figure 9 presents such plots for oscillators implemented using both buffered (figure 9*a*) and unbuffered (figure 9*b*) join gates. In both cases, the amplitude of the oscillations varies stochastically. In the buffered case, the period of oscillations remains constant until around 700 000 time units, at which point the population of buffered gates is exhausted and the oscillation breaks down. In the unbuffered case, however, the period of oscillation gradually increases as the gate population is depleted. This effect is certainly noticeable by around 700 000 time units, at which point around 70 per cent of the gates have been consumed. Gates are used up faster in the buffered case as there depletion is linear whereas in the unbuffered case it is exponential (see electronic supplementary material for the associated plots of gate populations). Thus, the kinetics of the buffered oscillator are constant right up until the oscillation fails, which implies that we could replace the lost buffered gates without affecting the kinetics of the oscillator, allowing the system to run for even longer time periods. This justifies the additional complexity of the buffered gate design from figure 7 compared with the simpler unbuffered design from figure 5.

We have also run simulations of the join gate and the three-phase oscillator using all four levels of abstraction and have examined the effect of leak reactions on the oscillator. In addition, we have carried out further comparisons of the buffered oscillator with an oscillator constructed using unbuffered reaction gates with garbage collection, which demonstrates that the different behaviour seen in figure 9 is not caused by the lack of garbage collection in the unbuffered gate design. We have also run longer simulations of the buffered oscillator to demonstrate that robust kinetics can be maintained over longer time periods than shown in figure 9. The reader is referred to the electronic supplementary material for further details.

### 3.3. Enabling leaks in the buffered join gate

Finally, we will demonstrate the analysis of unwanted interference in the buffered join gate design. When leak reactions are enabled under the *Infinite* semantics, the full CRN of the buffered join gate has 90 species and 468 reactions (of which 248 are leaks), compared with 22 species and 10 reactions without leaks. However, if we run a simulation with leaks enabled using the JIT compiler, we produce a CRN with far fewer species and reactions. The exact numbers vary because the JIT compiler only builds the CRN for the reactions which occur in a given stochastic simulation run, but averaging 10 JIT simulations only produced about 75 species and 125 reactions. This suggests that the majority of the leak reactions that are theoretically possible may never actually happen, owing to their low probability. Figure 8*b* shows an example plot from a buffered join gate simulation using the JIT compiler with leaks enabled. This is indistinguishable from the corresponding plot produced when all possible leak reactions are pre-computed. In general, we can use the simulator with the JIT compiler to save computation time and still obtain the exact dynamic behaviour of the full system (see electronic supplementary material).

However, comparing the two plots from figure 8 shows a qualitative difference between the behaviour of the system with leaks versus the system without leaks—when leaks are allowed, the population of output strands tends to keep growing over time, beyond the original population of 100 of each input strand. Looking through the set of possible reactions when leaks are included, we find a whole family of reactions similar to the following:


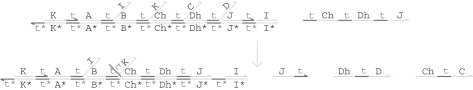


which consume buffered gates and erroneously produce the output strands <Ch t^∧^ C> and <Dh t^∧^ D>. These additional mechanisms for production of output strands explain how the populations of the output strands in the leak simulation turn out to be so much higher than the initial populations of the input strands. Since buffered gates can take part in these leak reactions, and there are many copies of the buffered gates, there are a large number of possible leak reactions that could occur and an even larger number of interleavings of these with the non-leak reactions. The situation is worse if there is a larger population of buffered gates, as this increases the probability of leak reactions occurring (we reduced the value of the Buffer variable from 1000 to 100 in the code from figure 6 to produce the plot in figure 8*b*). Furthermore, the CTMC becomes very large with leaks enabled, even for a relatively simple system such as a join gate. Analysing strand displacement systems with leak reactions is likely to be a major technical challenge: the problem is that leak reactions are not mediated by toehold domains, which greatly increases the complexity of the analysis.

## 4. Discussion

We have presented a hierarchy of semantic abstractions for modelling the behaviour of computational devices implemented using DNA strand displacement. The different semantic abstractions are suitable for different purposes, from high-level, simplified views for assembling large systems to low-level, detailed views for designing and verifying individual components of the DNA circuits. More complex models require more computational resources to simulate or analyse, so designers can move from simpler models to more complex models as their confidence in a new design increases. This means that we do not have to commit large amounts of computational resources to detailed analysis of a design until we have some degree of confidence that it will function as expected. Furthermore, our abstraction approach is general in the sense that it can be readily extended to define new abstractions that take into account new assumptions about the way in which species interact. Such rule-based modelling is becoming increasingly prevalent in the study of biochemical systems, for example [28,29].

The syntax presented in this paper extends the language described in Phillips & Cardelli [16] in a number of ways—we permit double-stranded complexes to be joined along either strand (not just the one which is oriented towards the bottom of the page) and also allow overhanging single strands along either strand. Along with these generalizations, we introduce the notion of rotation symmetry which overcomes some of the difficulties of conveniently representing nucleic acid structures on the page. In terms of the semantics, we have defined a new method for moving between different levels of abstraction, and have defined four distinct abstraction levels, coupled with new rules for handling leaks and unproductive reactions. We have also defined a method for JIT compilation of strand displacement systems, together with a method for generating a CTMC for further analysis. We have released a new version of the DSD language that incorporates these extensions. The close integration of modelling, simulation and analysis methods within a programming language environment is an important step towards the design automation of DNA strand displacement systems.

Our experience using these techniques to design the buffered join gate and three-phase oscillator shows how the DSD language can be integrated in the scientific workflow. We initially used the simplified *Infinite* level of abstraction to analyse the behaviour of a single buffered join gate, constructing the CTMC to explore all possible reduction paths and check for unwanted behaviour. We then moved to the more involved *Detailed* level of abstraction and performed a similar analysis, which required more computation time. We then switched back to the *Infinite* level of abstraction, so we could produce a tractable model of a three-phased oscillator, using several join gates as components. Owing to the larger populations involved in the three-phase oscillator, we had to switch to stochastic simulation as our primary means of analysing the behaviour of the system. We believe that this approach to modelling DNA interactions is a good fit for the natural workflow of the scientific design automation process.

To our knowledge, this paper is the first to present a formal, generic method for automatically deriving multiple levels of abstraction for a given DNA strand displacement system. As mentioned above, Zhang & Winfree [22] described abstracted models for the case of toehold exchange reactions—our contribution is to formalize and extend this approach to cover a wider range of systems. The gate designs presented in this paper employ a three-domain scheme for representing signals as single strands, which was introduced in Cardelli [25]. Soloveichik *et al.* [17] employed a more complex representation with additional toeholds in the signal strands. They also described a means of using DNA as a substrate to emulate arbitrary chemical kinetics. Their work can be viewed as complementary to the buffered gate scheme described in this paper, which is concerned with *maintaining* those kinetics for an extended period. Another scheme for representing signals is Cardelli's two-domain scheme [26], which only involves species with no overhanging strands. This enables new construction methods: one can construct two-domain gates by using enzymes to introduce breaks in a double-stranded complex, instead of annealing multiple single strands in solution. In principle, one could also construct buffered versions of these two-domain gates.

Switching between different semantic abstractions can qualitatively change the behaviour of systems. We demonstrated above that adding leak reactions significantly alters the behaviour of our join gate design, and many chemical oscillators are easily perturbed by the presence of leaks. Furthermore, certain programming idioms may not be possible under certain semantic abstractions. We mentioned above that unproductive reactions never appear when the *Infinite* level of abstraction is selected. In particular, the possibility of branch migration is not sufficient for a reaction to count as productive in this case (since species are considered equal if they differ only by branch migration steps). This means that co-operative displacement [30] cannot be modelled at the *Infinite* level of abstraction, because even though the first incoming strand may be able to perform branch migration steps, it does not stay bound long enough for a second strand to arrive and complete the co-operative displacement process. Thus, one must take care to select the correct semantic abstraction for a given program.

Our buffered reaction gate design addresses the issue of running strand displacement computations with quasi-constant kinetics over extended periods of time. By maintaining a large population of ‘buffered’ gates from which a smaller population of ‘initialized’ gates are drawn on demand, we separated the on-going reaction rates from the total population of gate structures in the system (provided that the buffer is periodically replenished before all the buffered gates are consumed). This separation is desirable for designing strand displacement systems with robust kinetics, however, there may be other ways of achieving this effect. One possibility is the use of remote toeholds [31], which insert a non-matching spacer between the toehold and the long recognition domain in both strands and gates. When a strand binds to the remote toehold, there is an internal diffusion step that produces a bulge, after which branch migration and strand displacement can proceed on the recognition domain. Genot *et al.* [31] demonstrated that modifying the design of the spacers allows precise control of the reaction rate. In particular, the internal diffusion step can be made rate-limiting, with the result that the reaction rate remains constant over a wider range of concentrations. However, this comes at the cost of considerably slowing down all binding reactions in the system.

Although the rules of the DSD language ensure that no secondary structures can be created, when physical DNA strands are mixed together, it may still be possible for them to form unwanted structures. This is because the DSD syntax introduced in §2.1. implicitly works at the level of interactions between complementary domains as opposed to interactions between individual nucleotides. While the domain abstraction is reasonable for the high-level design of strand displacement systems, when it comes to laboratory implementations of these designs, one must inevitably move to a lower level of abstraction. The DSD compiler currently checks for a number of interactions that could potentially create unwanted structures such as hairpins and rings, for example, when a gate folds back on itself, and signals an error to the user. However, more work is needed to ensure that species that behave as desired in DSD will also exhibit the same behaviour in the physical system, particularly with regards to branching structures. One way to achieve this is to generate the complete set of nucleotide sequences and to use tools such as NUPACK [32] and Multistrand^2^ to perform rigorous structural analysis at the nucleotide level. Ideally, in future, we would like to detect as many unwanted interferences as possible at the domain-level, directly within the DSD compiler. Thus, our work is complementary to existing work on low-level design of biomolecular computers, and indeed, we view DSD as but one component in a wider tool chain providing automation support for designers of strand displacement systems.

The DSD language presented above places no restrictions on the order in which domains may appear within a DNA strand. This means that it is permissible to have two or more neighbouring toeholds on the same strand. In this case, the binding reactions are computed as one might expect: a separate binding reaction for each toehold followed by a cover reaction that clamps down the remaining adjacent toeholds. Binding along consecutive toeholds is useful for modelling fast, irreversible binding between strands, for example, to model threshold binding as described in Qian & Winfree [33]. Since the binding strength increases exponentially with the number of bound bases, two consecutive toeholds will generally behave like an exposed long domain hybridizing with its complement, which circumvents our assumption that all interactions are mediated by reversible binding on a single toehold domain. Hence, we view programs that use multiple adjacent toeholds as a potentially unsafe subset of the DSD language, which we advise users to exploit with care. In future versions of DSD, we expect to provide a means of specifying the length of domains within the syntax of the language, which would allow such systems to be modelled in a more rigorous way.

Once a system has been rigorously specified, we have the possibility of verifying that it satisfies certain correctness properties. Probabilistic model checking offers great potential for verifying the correctness of DNA computing devices specified using the DSD language. Tools such as the PRISM model checker [34] can compute quantitative properties from a CTMC, such as the expected time to reach the terminal state. Model checkers can also verify that systems satisfy properties expressed in a temporal specification logic such as continuous stochastic logic (CSL) [35], for example, checking that the system must always pass through a particular intermediate state. However, model checking is currently only feasible for very small systems because the size of the CTMC grows exponentially as species populations increase. Our semantic hierarchy mitigates this to a limited extent because we can choose to model check at a simpler level of abstraction (e.g. *Infinite*), which produces fewer different species and reactions. Even so, model checking is limited in that it cannot prove anything about the behaviour of a component in context, which is key for the design of scalable systems. For this, we would need formal machine-assisted proofs of correctness.

Finally, the accuracy of our simulations is affected by our ability to correctly assign rates to the various reactions. In particular, we do not currently recompute rate constants between the four levels of abstraction. This means that the kinetics of a system may vary slightly depending on which semantic abstraction is chosen. This situation could be improved by integration with tools such as NUPACK and Multistrand, which would allow us to compute more realistic rate constants given the specific nucleotide sequences assigned to the domains in question, and by looking into ways to estimate more accurate rate constants for reactions at the different levels of abstraction. We could then use these values to produce more accurate models.

## Footnotes

1 A user manual for the tool is available online at http://research.microsoft.com/dna/manual.pdf.

2 http://www.dna.caltech.edu/DNA_Sequence_Design_Tools/.
